# Renal tumouroids: challenges of manufacturing 3D cultures from patient derived primary cells

**DOI:** 10.1007/s12079-022-00666-2

**Published:** 2022-01-31

**Authors:** Agata Nyga, Katerina Stamati, Patricia A. Redondo, Tayebeh Azimi, Andrew Feber, Joana B. Neves, Rifat Hamoudi, Nadège Presneau, Soha El Sheikh, Maxine G. B. Tran, Mark Emberton, Marilena Loizidou, Umber Cheema

**Affiliations:** 1 Research Department of Surgical Biotechnology, Division of Surgery & Interventional Science, University College London, London, UK; 2 Cell Biology Division, MRC Laboratory for Molecular Biology, Cambridge Biomedical Campus, Francis Crick Avenue, Cambridge, CB2 0QH UK; 3 Centre for Molecular Pathology, Royal Marsden NHS Trust, London, UK; 4 Specialist Centre for Kidney Cancer, Royal Free London NHS Foundation Trust, London, UK; 5 Sharjah Institute for Medical Research, College of Medicine, University of Sharjah, Sharjah, United Arab Emirates; 6 School of Life Sciences, University of Westminster, London, UK; 7 Cellular Pathology Department, Royal Free London Foundation Trust, London, UK; 8 Centre for 3D Models of Health and Disease, Research Department of Targeted Intervention, Division of Surgery & Interventional Science, University College London, Charles Bell House,43-45 Foley Street, London, W1W 7TS UK; 9 Department of Urology, University College London Hospitals NHS Foundation Trust, London, UK

**Keywords:** 3D cancer cultures, Patient derived 3D cultures, Renal cancer, Renal cancer mutations, Tumouroids

## Abstract

Recent advancements in 3D in vitro culture have allowed for the development of cancer tissue models which accurately recapitulate the tumour microenvironment. Consequently, there has been increased innovation in therapeutic drug screening. While organoid cultures show great potential, they are limited by the time scale of their growth in vitro and the dependence upon commercial matrices, such as Matrigel, which do not allow for manipulations of their composition or mechanical properties. Here, we show a straightforward approach for the isolation and culture of primary human renal carcinoma cells and matched non-affected kidney. This approach does not require any specific selection for cancer cells, and allows for their direct culture in amenable 3D collagen-based matrices, with the preservation of cancer cells as confirmed by NGS sequencing. This method allows for culture of patient-derived cancer cells in 3D microenvironment, which can be used for downstream experimentation such as investigation of cell–matrix interaction or drug screening.

**Supplementary Information:**

The online version contains supplementary material available at 10.1007/s12079-022-00666-2.

## Introduction

In vitro cell-line cultures have been invaluable tools to study the mechanisms and interrogate the molecular events leading to oncogenesis, tumour growth and aggressiveness. However, cancer cell heterogeneity means that capturing the range of cell specific responses is difficult using cell lines. For renal cancer, over 60% of somatic mutations are not detectable in all tumour regions, i.e. are not clonal, indicating significant tumour heterogeneity (Gerlinger et al. 2012), and highlighting the need for in vitro models that can recapitulate both patient and tumour heterogeneity.

Three dimensional (3D) in vitro cultures of human tumours go back to the late 70s with cultures in gelatin foam sponges and hollow fiber matrices producing glandular structures “organoids” (Rutzky et al. 1979). This was followed by cancer cell expansion through xenografting in mice (Köpf-Maier and Zimmermann 1991) to develop an organoid culture assay (OCA) to test drug sensitivity, the first 3D model to test individual tumours’ drug sensitivity and resistance in vitro - “anti-oncogram” (Köpf-Maier and Kolon 1992). It took another 15 years to develop improved methods to culture normal human epithelial organoids (Karthaus et al. 2014; Sato et al. 2011, 2009), and human tumour organoids, or tumouroids, without the need to first passage the cells in mice (Boehnke et al. 2016; Fujii et al. 2016; Gao et al. 2014; van de Wetering et al. 2015).

Renal cell carcinoma (RCC) incidence rates have been rising since the 1970s, partly due to increased use of imaging. Despite the advances in new immunotherapies for advanced disease, there has been no impact on overall survival, highlighting the need for personalised targeted therapy rather than a ‘one-size fits all’ approach. RCC is not a uniform malignant phenotype, and has many subtypes, including clear cell (ccRCC), papillary and chromophobe; each differing in relative aggressiveness, pattern of reoccurrence and in overall prognosis (Kuroda and Tanaka 2014). Renal cell carcinoma in vitro cultures using primary human cells or human tumour organoids are sparse. Early studies reported success rates of 12.7% in establishing continuous renal cancer cell lines (Ebert et al. 1990), while more recent studies show that primary RCC cells can be grown in vitro in a standard cell culture medium (Dulbecco’s modified Eagle’s medium with nutrient mixture F-12, or RPMI 1640) with supplementation of serum and human transferrin. Still, normal kidney cells showed greater proliferation than the primary tumour cells in 2D (Valente et al. 2011), and formation of 3D organoids–100% for normal kidney cells and 67% for ccRCC (Grassi et al. 2019). ccRCC 2D cultures (from Grade 2 and 3 tumours) were also established in serum-supplemented DMEM, with over 90% of cells expressing cytokeratin, vimentin and CD13, and over 60% of cells positive for carbonic anhydrase IX (CA9), a marker specific for ccRCC. These cultures also showed a strong correlation in genomic profiles with the tumour tissue from which they were derived (Cifola et al. 2011). On the other hand, metastatic RCC were cultured in 2D in CellGro SCGM medium, a specialized medium for expansion of haematopoietic and progenitor cells, with over 90% of cultured cells expressing CD10 (marker of certain RCC subtypes) (Dragoni et al. 2014). However, the limitations of the 2D cultures of primary human RCC cells is still reported to be their decline in proliferation and growth arrest after few passages. Addition of ROCK1 inhibitor (to prevent anoikis) during tumour dissociation steps significantly increased the clonogenic frequency and tumourigenic potential of the primary ex vivo ccRCC samples (Gedye et al. 2016), and was used to generate an organoid biobank of paediatric kidney tumours (Calandrini et al. 2020).

To address the challenges in studying primary human RCC in vitro, we describe a method of manufacturing 3D renal cancer masses (renal tumouroids) from cells isolated from patients with RCC. We took a novel approach, where we recapitulated the 3D matrix architecture and kidney tissue stiffness (Bensamoun et al. 2011). Isolated primary cells were both cultured in 2D conditions or in the 3D matrix composed of collagen type I supplemented with other matrix proteins. We used a patented and semi-automated process, based on our previously reported UCL patent (Brown et al. 2005) to compress the cell populated hydrogel and create dense tumouroids (Magdeldin et al. 2017; Nyga et al. 2013). We describe several challenges in terms of characterization of cells, evaluation of growth conditions, towards establishing a robust pathway from clinic to bench.

## Materials and methods

### Surgical tissue access and collection

Tissue samples (n = 24) were obtained with informed consent from patients undergoing nephrectomy at the Specialist Centre for Kidney Cancer, Royal Free Hospital NHS Foundation Trust (London, UK; REC reference number: 16/WS/0039). The following criteria were used for exclusion: cystic tumours, risk of compromising pathologic staging as judged by the pathology team, the patient having received chemotherapy, biologic targeted agents or radiotherapy less than 6 months before surgical intervention, and inability to provide informed consent. Information collected for each sample included: collection date, age, gender, date of diagnosis, association with hereditary syndromes, family history of 1st degree relative with renal cancer, clinical TNM staging, previous biopsy, and the presence of metastasis at diagnosis.

### Tissue handling

#### Tissue transfer

Samples were transported to the laboratory on ice in Wash Medium: RPMI (Cat# 21875091 ThermoFisher, Loughborough, UK) supplemented with: 100 units penicillin and 100 μg/ml streptomycin (Cat# P0781 Sigma-Aldrich, Gillingham, UK), 100 μg/ml gentamicin (Cat# G1272 Sigma-Aldrich), and 10 μg/ml Fungizone (Cat# A9528 Sigma-Aldrich) for immediate processing, or stored in Wash Medium at 4 °C until processed (24-48 h following surgery).

#### Primary cell isolation

Following removal of macroscopic fat and/or necrotic tissue, the sample was weighed and measured, and its gross morphology recorded. Tissues were finely minced with scalpel blades and digested with 1X collagenase/hyaluronidase (STEMCell Technologies, Cambridge, UK) in DMEM or with Tumour Dissociation Kit enzymes (4.7 ml of Wash Medium, 200 µl enzyme H, 100 µl enzyme R, 25 µl enzyme A, per 1g of tissue, Miltenyi Biotec, Surrey, UK) in a Petri dish. The dish was incubated for 60 min at 37 °C on a shaker, and samples were inspected and further minced as necessary.

The mixture was resuspended in 20 ml of Wash Medium (per 1 g of tissue), and filtered using 100, 70 and 40 µm cell strainers (Fisherbrand, Loughborough, UK). The filtrate was centrifuged at 300 g for 7 minutes. The cell pellet was resuspended in 24 ml of Wash Medium, and layered on top of 9 ml of Histopaque (Cat# 10771, Sigma-Aldrich) and centrifuged at 650 g, for 20 minutes. The buffy coat with cells was removed and washed with 20 ml of Wash Medium by centrifugation at 300 g for 7 min. Cells were resuspended in Wash Medium and counted using Luna-II Automated Cell Counter (Cat# L40002, LabTech, Heathfield, UK).

#### Separation of cell types

The mixed cell population obtained above underwent further separation of specific cell types. Separation was carried out for a subset of samples (n = 6) which yielded a high number of cells, to investigate both the presence of different cell types and to enrich the cancer cell population.

##### Fibroblast isolation

To positively select for fibroblasts, 10^6^ cells from the total isolate were resuspended in 80 µl of Sorting Buffer (0.5% bovine serum albumin (BSA) and 2 mM EDTA (Sigma-Aldrich) in PBS (ThermoFisher). 20 µl of anti-fibroblast beads (Cat# 130-050-601, Miltneyi Biotec) were added, mixed and incubated for 30 min at room temperature (RT). 500 µl of Sorting Buffer was added to the sample and mixed gently. Beads with positively selected cells were isolated using a LS column in a magnetic field (MidiMACS Separator, MACS MultiStand, Cat# 130-042-301, Miltenyi Biotec), collecting both non-labelled and labelled cells. Fibroblasts were plated in NUNC flasks (5 x 10^5^ cells per 75 cm^2^, Sigma-Aldrich) in Fibroblast Medium (Fibroblast Growth Medium 2, Cat# 23020, PromoCell, Heidelberg, Germany) with 100 units penicillin and 100 μg/ml streptomycin.

##### Endothelial cell isolation

To positively select for the endothelial population, the mixed cell population was resuspended in 60 µl of Endothelial Medium (EGM-2 MV medium, Cat# CC-3202, Lonza, Basel, Switzerland) containing penicillin/streptomycin, per 10^6^ cells and 20 µl FcR blocking reagent. 20 µl of anti-CD31 beads (Cat# 130-091-935, Miltneyi Biotec) were added, mixed and incubated for 15 min at 4 °C. The cell mixture was resuspended in 1 ml of Endothelial Medium and centrifuged at 300 g for 3 min. Beads with positively selected cells and non-labelled cells were isolated using a LS column in a magnetic field. CD31^+^ cells were plated at 5 × 10^5^ cells per 75 cm^2^on collagen-coated (0.1 mg/ml rat tail collagen type I in PBS, Cat# 60-30-810, First Link UK, Wolverhampton, UK) cell culture flasks in supplemented Endothelial Medium.

### Culture in 2D and 3D

#### 2D cultures

Cells not processed for specific subtype isolation (“Total cell isolate”) and non-labelled cells obtained following the bead-isolation steps (“Post-sorting cell isolate”) described above, were plated (NUNC plates, 5×10^5^ cells/75 cm^2^) in Cancer Medium (RPMI, ThermoFisher, supplemented with 10% Foetal Bovine Serum (FBS, ThermoFisher), and penicillin/streptomycin. Cells were incubated at 37 °C in a humidified atmosphere and 5% CO_2_. Medium was changed every 2 to 5 days. Cells were monitored under light microscopy every 2 to 3 days. If no cell growth was observed within 15 days or no further cell colonies formed at day 30, samples were disposed of. Cells were passaged at 70–80% confluency.

#### Spheroid culture

Total or Post-sorting cell isolate were tested for their spheroid forming ability. For this, cells were placed in low attachment cell culture plates (10^4^ cells/well, 6-well plate) and cultured in Spheroid Medium (Advanced DMEM/F12 (Cat# 12634-028, ThermoFisher) supplemented with 2 mM L-glutamine, 1 x N2 supplement (Cat# 17502-048, ThermoFisher), 1x B27 supplement (Cat# 17504-001, ThermoFisher), 1 mM N-acetyl-L-cysteine (NAC, Cat #A9165, Sigma-Aldrich), penicillin/streptomycin) or in STEM Cell Medium (Cat# 130-104-368, Miltenyi Biotec). Formation of spheroids was observed under light microscopy and images were taken to record spheroid size. If no spheroids were formed within 7 days, samples were disposed of.

#### Tumouroid culture

The 3D cultures were manufactured using the RAFT™ system (Real Architecture for 3D Tissue, Cat# 016-0R92, Lonza) as we previously described (Magdeldin et al. 2014). Briefly, cells (50–100, 000 cells per gel, or spheroids) were suspended on ice in a neutralised collagen type I solution (80% rat tail collagen type I, 2.05 mg/ml, 10% 10X Minimal Essential Medium, Cat# 21430020, ThermoFisher) with 50 µg/ml laminin (Cat# 734-1098, VWR, Lutterworth, UK) and cells in Cancer Medium (4.2%).

The cell-collagen-laminin mixture was immediately plated (240 µl/well in 96-well plates) and incubated for 15 min at 37 °C to allow for collagen polymerization. After polymerization, to increase collagen density, gels were compressed by placing absorbers on the top surface of each, to remove liquid (RAFT 3D system, Lonza) for 15 min at RT. Following this, absorbers were removed, and Cancer Medium or Spheroid Medium was added (200 µl per well). Tumouroids were incubated at 37 °C in a humidified atmosphere and 5% CO_2_. 50% the medium was refreshed every 2 days.

### Immunofluorescence

Tumouroids were fixed in 150 µl of 10% neutral buffered formalin (NBF) for 30 min at RT. Non-specific binding was blocked with 200 µl of Blocking Buffer (1% BSA, 0.3% Triton X-100 in PBS) for 1 h at RT. Tumouroids were incubated overnight in 100 µl of primary antibody (Table 1) diluted in Blocking Buffer at 4 °C. Secondary antibody diluted in Blocking Buffer was incubated for 2 h at RT (covered from light). Samples were mounted in medium with DAPI (Vectashield) and imaged using inverted fluorescent microscope (EVOS FL Cell Imaging System, ThermoFisher).

**Table 1:** Primary antibodies (Abcam) used for fluorescent imaging

Antibody	Type	Species	Localisation	Dilution	Cat #
CK8	Primary	Mouse	Membrane, Cell junction	1:200	ab9023
CD31	Primary	Rabbit	Cytoplasm	1:200	ab9498
Vimentin	Conjugated	Mouse	Membrane & cytoplasm	1:1000	ab195877
Anti-Mouse	Secondary	Goat/Donkey		1:500	ab150113/ab150108
Anti-Rabbit	Secondary	Donkey		1:500	ab150077

### Fluorescence-activated cell sorting (FACS)

Cancer cell monolayers were gently detached from 2D tissue culture dishes using Tryple Select enzyme (4 ml per 75cm^2^, Cat# 12563-011, ThermoFisher). Firstly, cell suspension (5 x 10^5^ cells) was stained for live and dead cells using Live/Dead™ Fixable stain kit (Near-IR, Cat# L10119, ThermoFisher), followed by blocking the Fc receptor (Cat# 564220, BD Biosciences, Wokingham, UK) in stain buffer (1% BSA, 0.09% sodium azide in PBS) for 10 min at RT. Cells were stained in 100 µl of stain buffer and fluo-labelled antibody or isotype control (Table 2, antibodies concentrations were optimized using renal carcinoma cell lines CAKI-2 and ACHN, human endothelial cell line HUVEC, human dermal fibroblasts HDF and monocytic cell line U937) for 1 h at RT. Following staining, cells were fixed in 250 µl Cytofix fixation buffer (Cat# 554655, BD Biosciences, Berkshire, UK) for 15 min at 4 °C. For intracellular staining, fixed cells were permeabilized in 1 ml of ice-cold Phosflow Perm buffer III (Cat# 558050, BD Biosciences) for 30 min on ice. Permeabilised cells were incubated for 1 h at RT in 100 µl stain buffer with intracellular marker (vimentin, CK-7/-8) antibody or isotype control. Cells resuspended in 1 ml of staining buffer were assessed on a cell analyzer (BD LSRFortessa™, BD Biosciences) using BD FACSDiva software (version 6.2).

**Table 2:** Primary antibodies used for single cell analysis

Antibody	Fluorochrome	Dilution	Positive control	Negative control	Cat #	Company
CK-7/-8	Alexa Fluor 647	1:100	ACHN/CAKI-2	HDF/HUVEC	563,614	BD Biosciences
CD44	APC	1:100	U937/ACHN	HDF	560,890	BD Biosciences
CD45	BV510	1:100	U937	HUVEC/HDF	563,204	BD Biosciences
CD31	Alexa Fluor 647	1:100	HUVEC	HDF	561,654	BD Biosciences
CD105	BV421	1:100	U937	HDF	563,920	BD Biosciences
Vimentin	Alexa Fluor 488	1:100	HDF	U937	562,338	BD Biosciences
CA9	PE	1:100	ACHN/CAKI-2	HDF/HUVEC	FAB2188P	R&D Systems
iso	PE	1:100			554,680	BD Biosciences
ISO	BV510	1:100			562,946	BD Biosciences
iso	Alexa Fluor 647	1:100			557,714	BD Biosciences
iso	Alexa Fluor 488	1:100			557,721	BD Biosciences
iso	APC	1:100			555,745	BD Biosciences
iso	BV421	1:100			562,438	BD Biosciences

### DNA extraction from fixed tumouroids

DNA was extracted from tumouroids fixed in 10% NBF using QIAamp DNA FFPE kit (#56404, Qiagen, Manchester, UK) according to manufacturer’s instructions. Briefly, 3–4 tumouroids from same patient were pooled together in 1.5 ml microtube and resuspended in 180 µl Buffer ATL, followed by incubation for 30 min, addition of 20 µl of proteinase K and quick vortexing. Samples were incubated for 2 h at 56 °C under rotation (500 rpm) to allow complete lysis, followed by 1 h at 90 °C. Additional 200 µl of Buffer AL was added and sample was mixed by vortexing, followed by addition of 200 µl pure ethanol and mixing by vortexing. Lysate was then transferred onto the QIAamp MinElute column in a 2 ml collection tube, centrifuged at 6000 x g for 1 min, followed by washes with Buffer AW1 and AW2. Membrane was then left to dry and DNA was eluted using 20–100 µl ddH_2_0. DNA integrity and quantification was measured using the TapeStation 2200 (Agilent) platform and the High Sensitivity D1000 Screen Tape, according to manufacturer’s instructions.

### Genomic analysis with next generation sequencing

NGS libraries were prepared from 50 to 400 ng of DNA using the KAPA HyperPlus Kit (Kapa Biosystems, Wilmington, MA, USA) and IDT UDI 8 bp adapters (Integrated DNA Technologies, Coralville, USA), following the manufacturer’s protocol, including dual-SPRI size selection of the libraries (250–450 bp). To optimise enrichment and reduce off-target capture, pooled, multiplexed, amplified pre-capture libraries (up to 20 samples per hybridization) were hybridized overnight using 1 µg of total DNA to a custom design of DNA baits complementary to the genomic regions of interest (NimbleGen SeqCap EZ library, Roche, Madison, WI, USA). Hybridised DNA was PCR amplified and products purified using AMPure XP beads (Beckman Coulter, Danvers, MA, USA) and quantified using the Qubit dsDNA High Sensitivity Assay Kit with the Qubit 3.0 fluorometer (Invitrogen, Carlsbad, CA), and High Sensitivity D1000 TapeStation (Agilent, Santa Clara, USA).

Sequencing was performed on a NovaSeq6000 with 150bp paired-end reads and v1.5 chemistry, according to the manufacturer’s instructions. NovaSeq (Illumina, San Diego, CA, USA) runs were analysed using an in-house pipeline. For the demultiplexing bcl2fastq (v2.19) was used to isolate reads for each sample. The reads were aligned to the reference genome build GRCh37/Hg19 using Burrows-Wheeler Aligner (BWA-MEM), followed by the marking of PCR duplicates and calculation of various quality control (QC) metrics using Picard software (v2.21.1). Copy number was estimated by generalizing the coverage expected for a copy of any given gene, taking the average coverage across all target regions to estimate the average coverage of one target region. Any ratio below 0.5-fold was defined as a potential deletion, whereas a ratio above 2.4 was flagged as a potential amplification if 80% of the target regions had exceeded the thresholds. Manta (v0.29.6) was used for the detection of structural variants. Genome Analysis Toolkit (GATK) was used for realigning around indels to improve indel calling and base quality score recalibration for adjusting systematic errors made by the sequencer when estimating quality scores of each base call. Finally, GATK was also used for variant calling using HaploType Caller for tumour only analysis (limit of detection ~10%) and MuTect2 for tumour paired analysis. VCF files from unpaired samples were annotated using Illumina Varinat Studio v3.0, and the variants checked manually on IGV using different allele frequency plots.

### Statistical analysis

Quantitative results are expressed as mean ± standard error of the mean. Statistical analysis was performed using the Mann-Whitney U-test and graphs were prepared using GraphPad. A *p* < 0.05 was considered statistically significant.

## Results

Patients’ samples used in this study are listed in Table 3. We collected renal tumour samples, and matched non-affected kidney where possible, from 24 patients, which following histopathological analysis were identified as clear cell (ccRRCC) (58%), papillary RCC (21%), chromophobe RCC (4%), oncocytoma (13%) and urothelial cell carcinoma (4%).

**Table 3:** Patient and sample data

Type	Clear cell	Papillary	Chromophobe	Oncocytoma	Transitional cell
Number	58% (14/24)	21% (5/24)	4% (1/24)	13% (3/24)	4% (1/24)
Gender (M:F)	7:7	5:0	0:1	2:1	0:1
Age average (range)	57 (43–76)	58 (49–68)	66	74 (69–79)	75
Range of sample weight (g)	0.3–8.6	0.3–8.0	4.2	0.2–1.0	1.3
Range of total cells isolated	0.16–17.4 × 10^6^	0.11–10.8 × 10^6^	7.6 × 10^6^	2.63–8.97 × 10^6^	10.75 × 10^6^
2D cell growth	58% (7/12) 2*	100% (5/5)	*	100% (3/3)	100% (1/1)

*Cells frozen following isolation

In this study, only samples from ccRCC were included. ccRCC samples were further classified (Table 4) based on their nuclear grading using the Fuhrman classification into grade 1 (7%), grade 2 (36%), grade 3 (36%) and grade 4 (21%). From the isolated cells, 10 out of 12 samples (83%) successfully expanded in 2D *in vitro* cell culture, but only 5 out of 9 samples (56%) could be passaged beyond 2 passages, with those samples being mainly of Grade 3 and Grade 4.

**Table 4:** Growth of Clear Cell Renal Cell Carcinoma according to nuclear grading

Fuhrman Nuclear Grade	1	2	3	4
Number	7% (1/14)	36% (5/14) *	36% (5/14)*	21% (3/14)
2D cell growth (p0-p1)	100% (1/1)	75% (3/4)**	75% (3/4)	100% (3/3)
2D cell growth p2 >	0% (0/1)	50% (1/2)	33% (1/3)	100% (3/3)
Spheroid formation	100% (1/1)	33% (1/3)	100% (1/1)	0% (0/2)
Direct 3D culture	N/A	0	1	2
Indirect 3D culture	Spheroid in 3D	Spheroid in 3D (1) Cells in 3D (1)	Spheroid in 3D (1) Cells in 3D (1)	Cells in 3D (3)

*One sample cells frozen following isolation

**One sample had infection in the culture

**Fig. 1:**
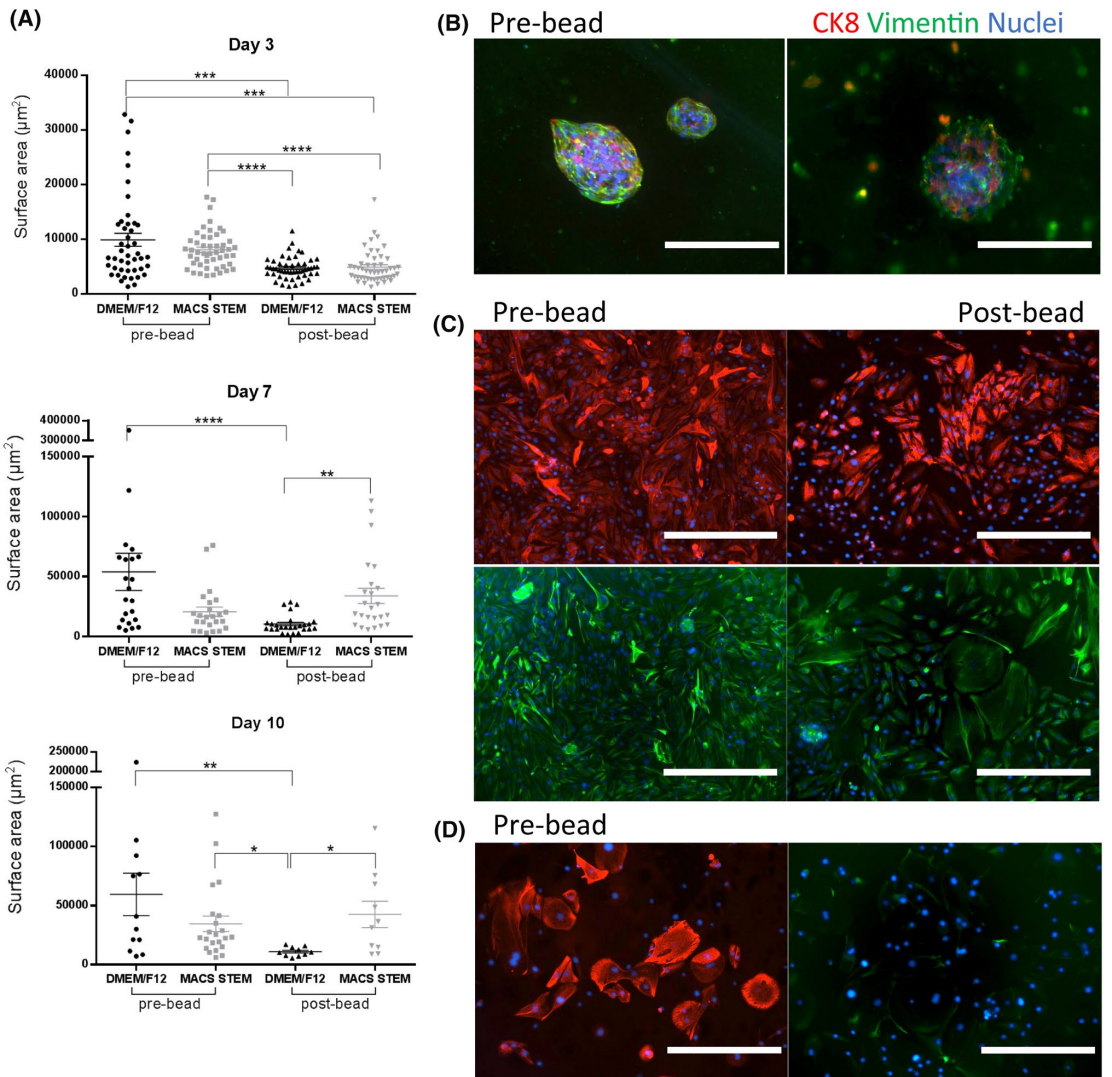
Comparison of pre-sorted and post-sorted clear cell renal cell carcinoma isolated cell populations. **a** ccRCC showed poor formation of spheroids (3 out of 7 samples). Here is an example of successfully grown spheroids from pre- (pre-bead) and post-sorting for stromal cells (post-bead) cultured in a Spheroid Medium (DMEM/F-12) or a commercial Stem Cell Medium (MACS STEM). Their surface area (µm^2^) was measured on day 3, 7 and day 10 **b** cultured spheroids were embedded in stiff collagen matrix and remained viable for up to 21 days, here fixed and stained for vimentin (Green) and CK8 (red) and nuclei (Blue) from pre-bead population cultured in Spheroid Medium (scale bar 400 µm). **c** cell samples grown in 2D from Grade 2 tumour showed positive CK8 and vimentin expression in both pre-bead and post-bead cell populations (scale bar 400 µm). **d** cells isolated from Grade 4 (pre-bead) had enlarged cell morphology, clear cell-like, with strong CK8 expression and weak vimentin expression (scale bar 400 µm)

We tested 2 methods to culture cancer cells. Either as mixed, non-sorted cell cultures or as sorted cultures where the stromal subpopulations (fibroblasts and CD31^+^ cells, Supplementary Figure 1) were cultured separately. Cancer cells from 7 patients from either sorted or mixed non-sorted cell isolates were first cultured in low-attachment plates to observe their ability to form spheroids (Figure 1). Spheroids formed in only 3 of the 7 samples and this was independent of cell sorting. However, we observed a significant increase in spheroid size in non-sorted cell population when compared to the sorted cell population (Figure 1a). Furthermore, when comparing different cell culture mediums for spheroid formation, we observed no significant difference between the two media used, either Spheroid medium or Stem Cell Medium, for each cell population. Culturing the formed spheroids in stiff collagen for up to 21 days maintained their integral structure, viability, and expression of both CK8 and vimentin (Figure 1b).

Culturing the sorted cell populations (fibroblasts, CD31^+^ and remaining cancer cells) in specific media did not result in specific outgrowth of any of these cell subpopulations. All cells were positive for CK8 and vimentin, and showed similar morphology to the cells from mixed, non-sorted populations (Figure 1c, Supplementary Figure 1–2). For further experiments, morphology, and expression of CK8 and vimentin changed according to the original tumour grade (Figure 1d). Cells from Grade 4 tumour showed heterogenous expression of CK8, with some cells strongly positive and some cells not expressing CK8, while vimentin expression decreased in all cells compared to cells grown from lower grade samples.

To confirm that our methods allow the culture of cancer cells from mixed cell populations, where the morphology of cells indicated a ccRCC phenotype, and exclude exclusive overgrowth of non-malignant epithelial or stromal cells, we assessed the presence of cancer cell surface markers using FACS and cancer mutations using Next Generation Sequencing (NGS).

**Fig. 2:**
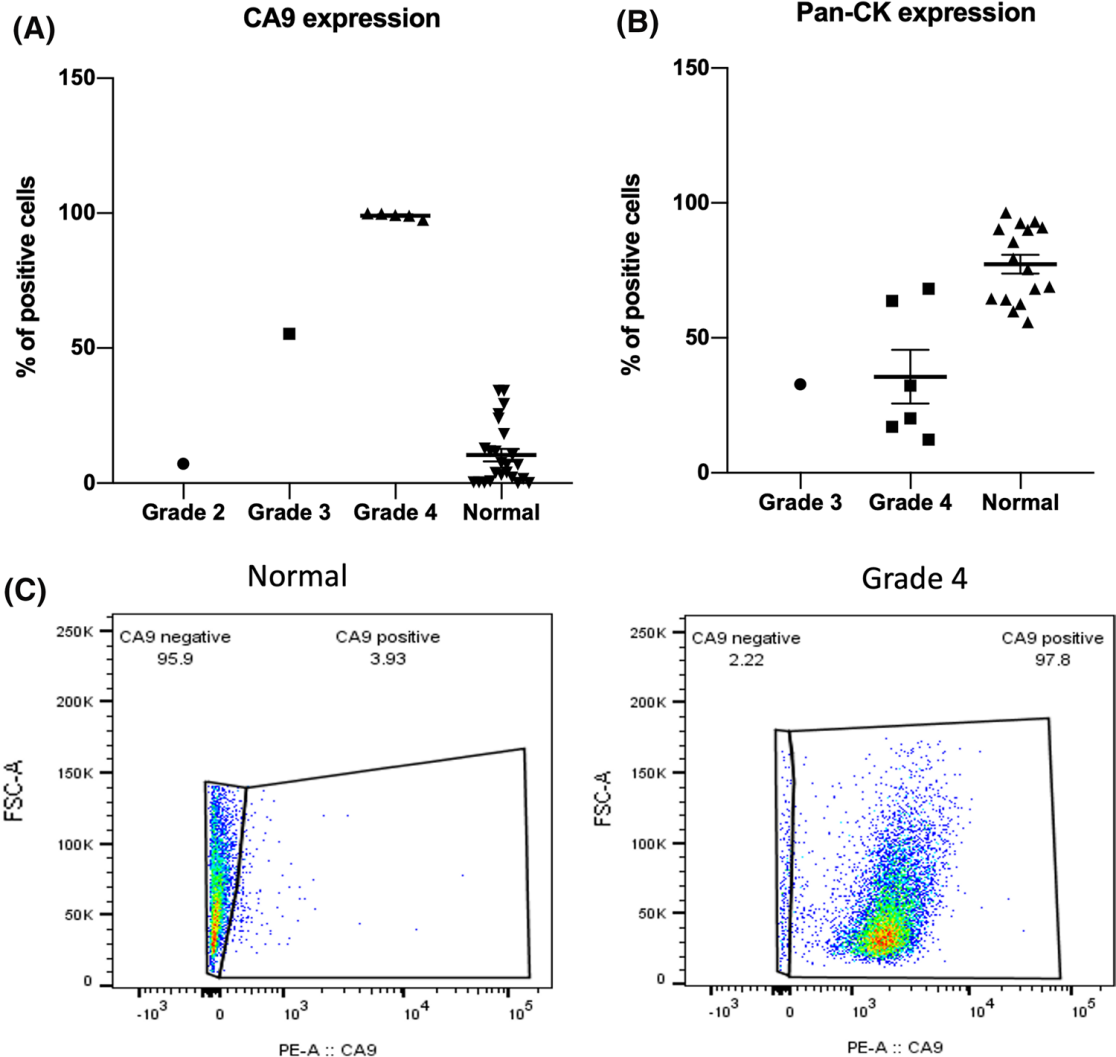
Single cell analysis of expression profile for CA9 and cytokeratin in ccRCC samples. CA9 was highly expressed in ccRCC cells from Grade 3 and Grade 4 samples, while low expressions were observed in Grade 2 and non-malignant samples (**a**), pan-cytokeratin was highly expressed in cells from normal samples, while the expression varied in samples from Grade 3–4 samples (**b**), comparison of CA9 expression between normal and Grade 4 sample (**c**)

To distinguish specific cell populations, we looked for the presence of the ccRCC marker carbonic anhydrase IX (CA9) by FACS. We found a higher percentage of CA9^+^ cells with increasing tumour grade, no CA9^+^ cells were found in matched healthy tissue (Fig. 2a,c). We also found that the number of cytokeratin (CK)^+^ cells was reduced in tumour samples compared to matched healthy tissue (Fig. 2b).

**Fig. 3:**
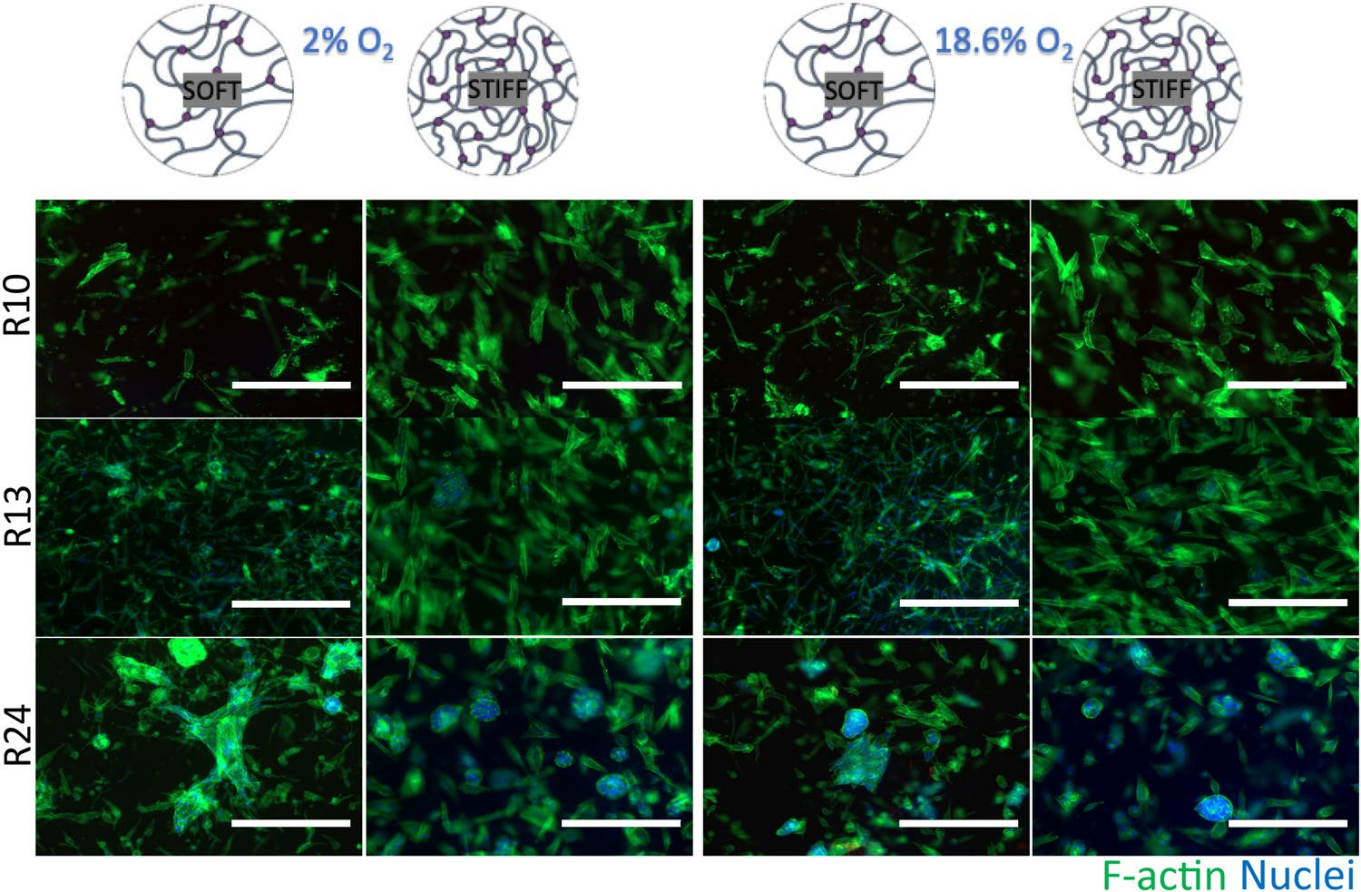
Non-malignant kidney cells morphology in collagen-based scaffolds. Non-malignant kidney epithelial cells (n = 3) were grown in soft or stiff collagen-based scaffold for 14 days under normoxia or hypoxia. Scale bar 400 µm

We further cultured freshly isolated malignant and non-malignant epithelial kidney cells in soft and stiff matrices under either normoxia or physiological hypoxia (2%) for 14 days. We found that non-malignant epithelial renal cells grew in both soft and stiff matrices under both hypoxia and normoxia. However, in soft matrices cells formed more elongated thin networks, while in stiff matrices, they arranged in sheet like structures, and could even form spheroid-like structures (Fig. 3).

**Fig. 4:**
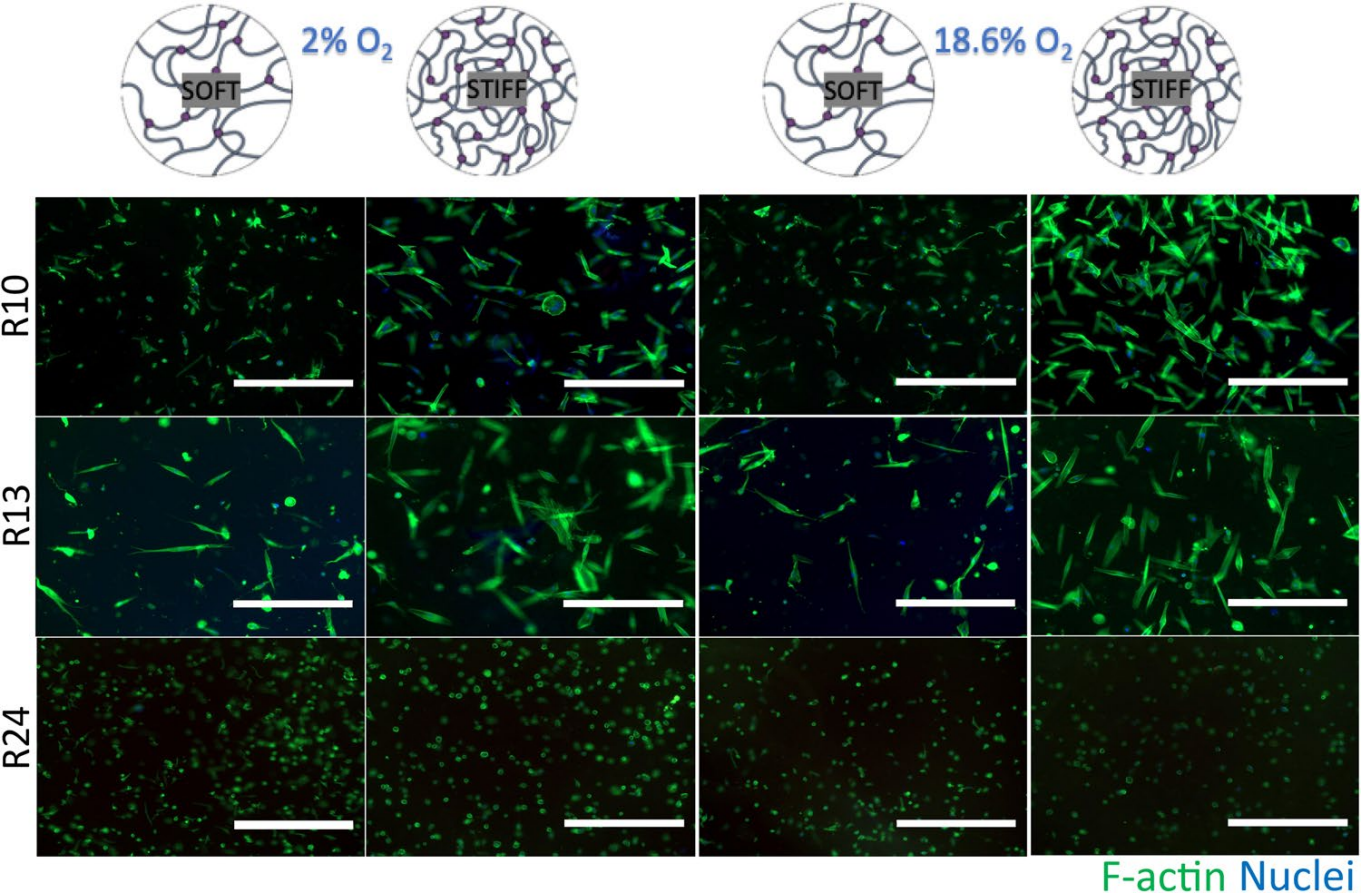
ccRCC cells morphology in collagen-based scaffolds. ccRCC cells (n = 3) were grown in soft or stiff collagen-based scaffold for 14 days under normoxia or hypoxia. Scale bar 400 µm

On the other hand, cancer renal cells showed similar morphology independent of the matrix stiffness or oxygen concentration (Fig. 4).

**Fig. 5:**
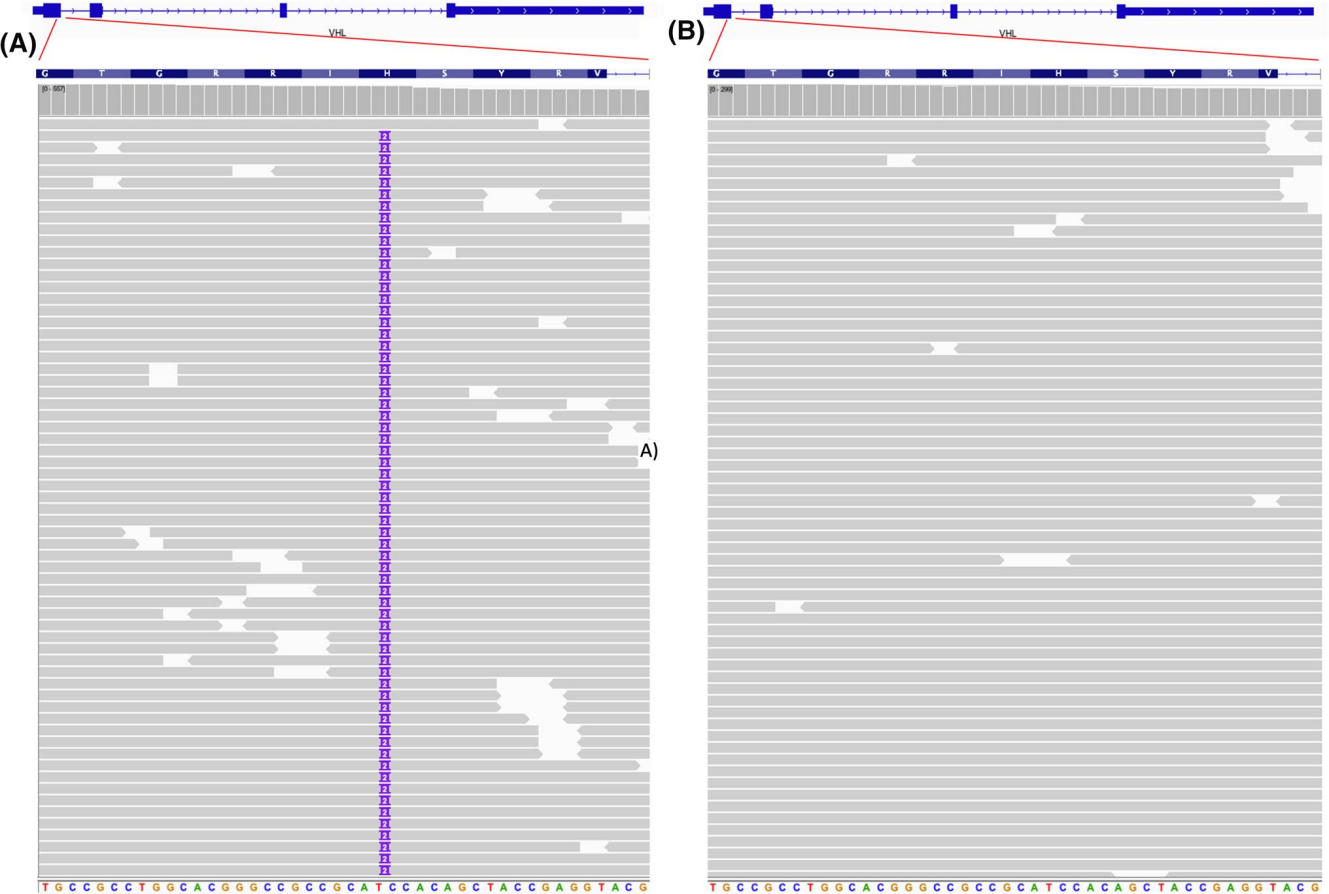
Snapshots of the genetic analysis of VHL-1 gene in tumouroids grown from renal tissues from patients R24. **a** R24, tumour tissue: I109*InsCC, **b** R24, matched normal tissue. The 2-base pair inseration is indicated by **2**

Finally, we used targeted NGS to identify the presence of RCC specific mutations, such as VHL, or other cancer specific alterations in samples from different tumour grades and matched healthy tissue which had been grown in the stiff 3D cultures. Cancer specific alterations were identified in all tumour samples compared to their corresponding normal tissue. This includes classical RCC alterations, such as 2pb duplication in the first exon of VHL(c.327_328dupCC) in case of R24 (Grade 4 ccRCC) (Fig. 5), previously associated with poor overall survival and resistance to therapy in RCC, and the likely driver in this tumour. Alterations were also identified in the KMT2C and KMT2D (R13–Grade 3 ccRCC) and FGFR1 (R10–Grade 2 ccRCC).

## Discussion

Kidney cancer encompasses a variety of histological subtypes. Our collection of post-nephrectomy tumour samples resulted in samples of clear cell RCC (58%), papillary RCC (21%), chromophobe RCC (4%), oncocytoma (13%) and urothelial cell carcinoma (4%). The efficacy in establishing organoid cultures differs depending on the renal carcinoma subtype. For paediatric kidney tumours, the efficacy was 75% for Wilms tumours, 100% for malignant rhabdoid tumours, and 75% for renal cell carcinomas, while non-malignant kidney had 100% efficacy (Calandrini et al. 2020). For this study we focused on samples from clear cell renal cell carcinoma (ccRCC) of different grades as they were the most frequent and to allow comparability results. The efficacy of 2D expansion was 83%, but long-term culture was only established in 56% of samples, mainly of Grade 3 and Grade 4. Cells isolated from the adjacent non-malignant part of the kidney had 100% efficacy in 2D growth. This highlights early cancer vulnerabilities upon dissociation from tissue and its microenvironment. Previously, limitations in primary cancer cell culture were reported to involve overgrowth by tumour-associated spindle cells or normal epithelial cells (Gao et al. 2014), however, in our study, culturing non-sorted or sorted populations enriched for cancer cells, resulted in similar growth of cancer cells in 2D and spheroids. ccRCC organoids in Matrigel were shown previously to lose their ability to form cohesive structure in long-term (several weeks) (Grassi et al. 2019), while we showed that the integrity of cancer spheroids can be maintained in stiff 3D collagen matrix for up to 21 days. Additionally, cells that were specifically selected during isolation using fibroblast or endothelial cell markers, did not expand as the selected subpopulations, but instead were positive for both CK8 and vimentin. ccRCC cells have been shown to display mesenchymal characteristics (Landolt et al. 2017; Sugimoto et al. 2016), whilst also showing highly vascularised phenotype (Qian et al. 2009; Yao et al. 2007) with a preference to endothelial like conditions in vitro and a display of vascular mimicry (Serova et al. 2016). Therefore, it is very likely that the specific cell sorting of stromal cells is not an appropriate approach to separate heterogenous ccRCC population as the cancer cells may express the same markers. While the higher grade ccRCC cancer cells showed distinctive morphology characteristic of their cancer type–enlarged cells with clear cytoplasms (Muglia and Prando 2015), the lower grade cells showed morphology similar to the epithelial cells isolated from normal samples. Previous work in this area has highlighted the potential for normal primary epithelial cells to overgrow primary cancer cells in vitro (Kodack et al. 2017). Our FACS analysis showed increase in CA9 positive cells–marker of ccRCC–with samples from higher tumour grade, confirming presence of ccRCC cells.

Isolated cells were also successfully cultured in soft and stiff 3D collagen-based matrices under either hypoxia or normoxia. Previously, ccRCC cultured directly in Matrigel following isolation, grew only as single cells, though their CA9 expression was still high (Na et al. 2020). In our conditions non-malignant epithelial kidney cells formed either elongated thin networks (soft matrix) or sheet-like structure and spheroid-like structures (stiff matrix), while cancer cells had no difference in growth between soft and stiff cultures with similar disperse morphology. NGS analysis of cancer and non-malignant cells in stiff matrices, confirmed presence of cancer cells, with mutations varying between the analysed samples. This supports the use of 3D collagen-based matrices for direct culture of freshly isolated non-malignant and cancer cells.

There is ongoing need for personalised medicine and for improved disease models to mimic disease and patient heterogeneity. One of the key challenges is to extract cancer cells and maintain them in culture without affecting their viability or phenotype. Extensive 2D cell expansion can lead to clonal selectivity, and acquisition of phenotypic changes, prior to any relevant experimental testing. The ability of growing freshly isolated cancer and normal cells directly in 3D allows for studying the cells already in correct microenvironment without causing any alterations due to a 2D cell culture. In this paper, we present an approach to isolate clear cell renal cell carcinoma cells from tumours of various grades. Along the process, we isolated non-malignant kidney epithelial cells from regions outside of the cancer margins. We showed that the cancer cells and kidney epithelial cells do not need selection or sorting, and through mechanical and chemical dissociation of tissue and culture of the isolated single cells in specific cell culture medium, we obtained cell cultures of interest. Furthermore, the cancer cells maintain their phenotype and genotype (CA9 expression, presence of specific ccRCC mutations) and can grow in 3D collagen-based matrices. While normal epithelial cells grow in both soft and stiff matrices, cancer cells show preference to stiffer gels–resembling the stiffer tumour microenvironment in vivo. This approach provides tools for further specialization of the 3D collagen scaffold to mimic closer the tumour microenvironment of choice–by addition of other relevant extracellular matrices or changing its stiffness.

## Supplementary Information

Below is the link to the electronic supplementary material.

Morphology of cells following magnetic sorting for fibroblast and CD31+ subpopulations and cultures in specific conditions. Morphologically cells have epithelial-like morphology (elongated but rounded) in both conditions. Scale bar 400 µm. (TIFF 8778 KB)

Kidney cells isolated and cultured from non-malignant samples. Kidney cells showed epithelial morphology with expression of CK8 and weak expression of vimentin (scale bar 400 µm). (TIFF 4306 KB)

## Abbreviations

2D: Two dimensional
3D: Three dimensional
CA9: Carbonic anhydrase IX
ccRCC: clear cell renal cell carcinoma
CK8: Cytokeratin 8
FACS: Fluorescence-activated cell sorting
FBS: Foetal bovine serum
OCA: Organoid culture assay
RCC: Renal cell carcinoma
