# Supplementary figures and images for: Renal tumouroids: challenges of manufacturing 3D cultures from patient derived primary cells
Source: J Cell Commun Signal. 2022 Jan 31;16(4):637–48. doi: 10.1007/s12079-022-00666-2 (PMC9733748; doi:10.1007/s12079-022-00666-2)

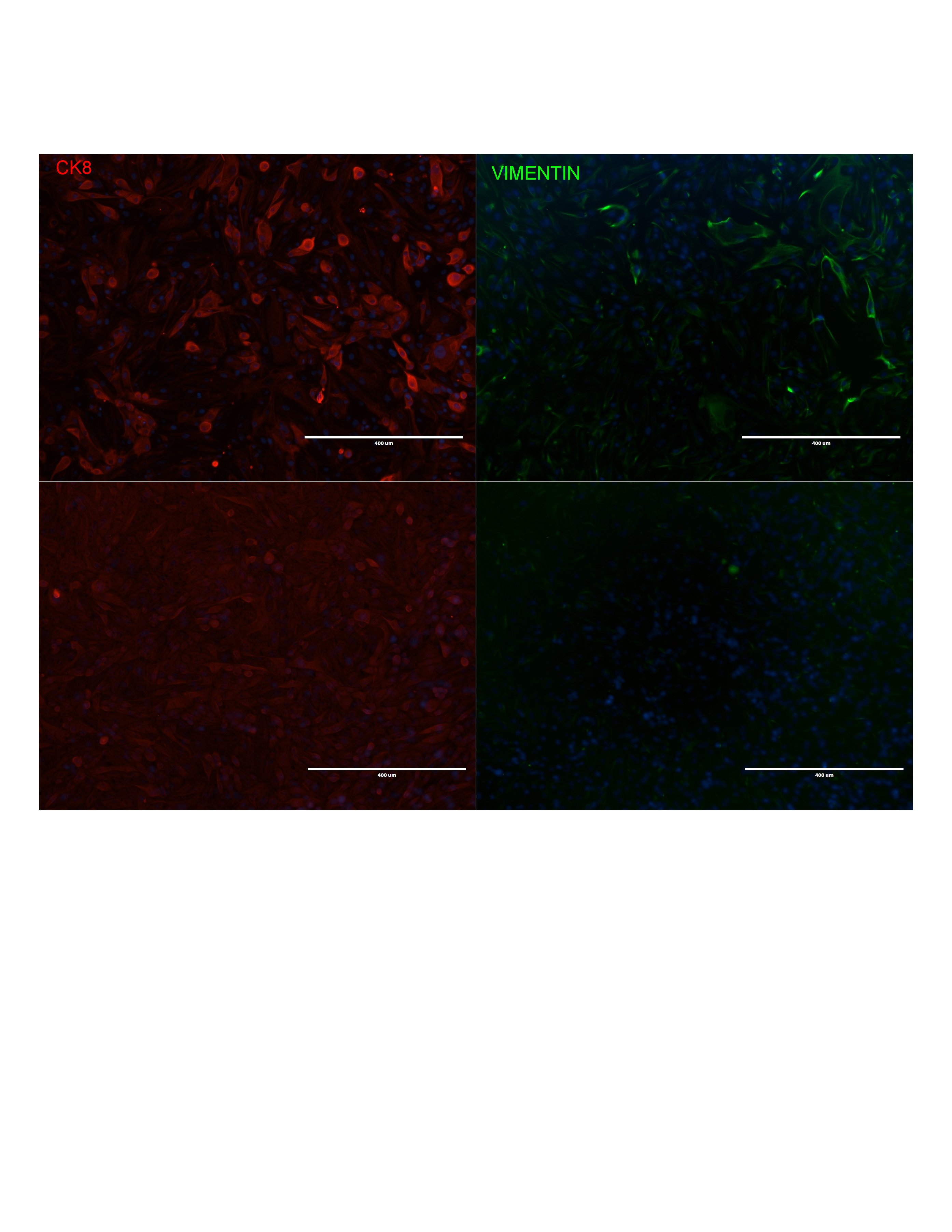

Supplement: Supplementary file 1 — Morphology of cells following magnetic sorting for fibroblast and CD31+ subpopulations and cultures in specific conditions. Morphologically cells have epithelial-like morphology (elongated but rounded) in both conditions. Scale bar 400 µm. (TIFF 8778 KB) [file 12079_2022_666_MOESM1_ESM.tiff]

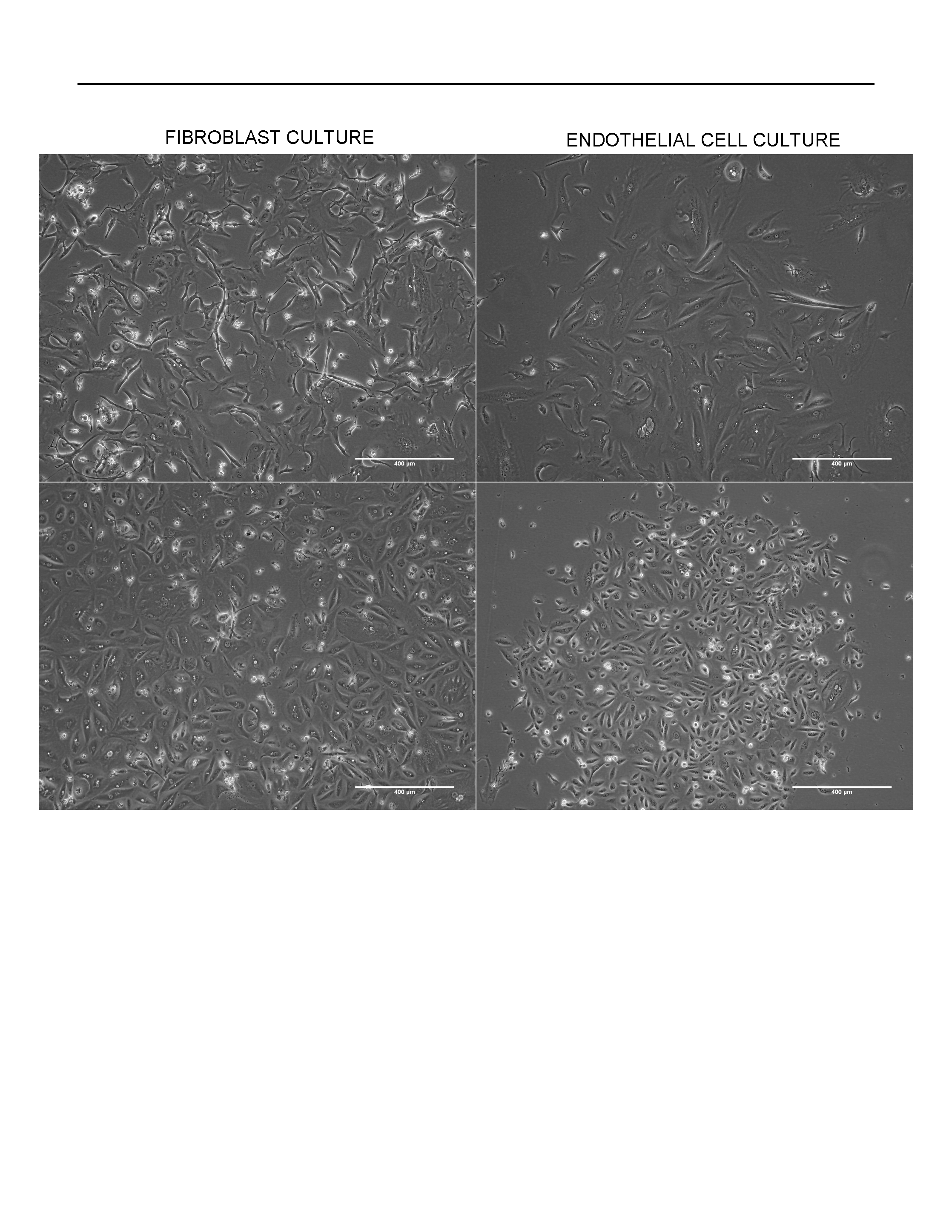

Supplement: Supplementary file 2 — Kidney cells isolated and cultured from non-malignant samples. Kidney cells showed epithelial morphology with expression of CK8 and weak expression of vimentin (scale bar 400 µm). (TIFF 4306 KB) [file 12079_2022_666_MOESM2_ESM.tiff]
